# The Role of CTLA-4 Exon-1 49 A/G Polymorphism and Soluble CTLA-4 Protein Level in Egyptian Patients with Behçet's Disease

**DOI:** 10.1155/2014/513915

**Published:** 2014-06-02

**Authors:** Sahar M. Abdel Galil, Hoda A. Hagrass

**Affiliations:** ^1^Rheumatology & Rehabilitation Department, Faculty of Medicine, Zagazig University, Zagazig 44511, Egypt; ^2^Clinical Pathology Department, Faculty of Medicine, Zagazig University, Zagazig 44511, Egypt

## Abstract

This study analyzed the association of the A/G SNP at position +49 of exon-1 in the CTLA-4 gene to the susceptibility and clinical manifestations of Behcet's disease (BD). It was performed on 60 Egyptian BD patients and 95 age- and sex-matched healthy controls. The genotypes for the +49 A/G polymorphism of the CTLA-4 gene were determined by PCR-RFLP, while the serum level of CTLA-4 protein was measured by ELISA. CTLA-4 +49 A allele (*P* < 0.001, OR = 3.084, and CI (95%) = 1.90–4.99) and A/A genotype (*P* < 0.001, OR = 6.643, and CI (95%) = 2.58–17.10) frequency distribution was significantly more increased in patients than in the controls, with no significant differences between males and females with regard to the genotype or allele frequency distribution. A/A genotype was associated with a more reduced expression of sCTLA-4 protein in patients than in the controls (1.76 ± 0.19 versus 1.91 ± 0.30, resp; *P* < 0.0007). In addition, it is associated with the occurrence of ocular and vasculitic manifestations of BD in the patient group. The CTLA-4 gene could be considered as a susceptibility and a disease-modifying gene to BD in Egyptian population that needs further confirmatory studies on larger cohorts.

## 1. Introduction 

Behçet's disease (BD) is a chronic, relapsing, inflammatory disorder affecting all sizes of arteries and veins. It is characterized by recurrent painful oral and genital ulcers and ocular and skin lesions. Arthritis, gastrointestinal, and central nervous system affection can also occur in BD [1].

Although the etiology of BD is not fully understood, it has been suggested to occur as a result of an interaction between a susceptible genetic background and various environmental factors that trigger an autoimmune process in genetically predisposed individuals [2, 3].

Although the majority of patients with BD are sporadic cases with no family history, the presence of BD patients' familial aggregation has long been noted [4–6]. The cytotoxic T lymphocyte-associated antigen- (CTLA-) 4 molecule is a 40 kDa transmembrane glycoprotein expressed on resting and activated T cells and nonlymphoid cells [7]. Along with the related CD28 costimulatory molecule, it regulates T-cell activation [8]. Both molecules bind to the same ligands, B7–1 (CD80) and B7–2 (CD86), although CTLA-4 binds with a much higher affinity [9].

CTLA-4 protein plays an important role in downregulating T-cell activation and effector function, in part, by inhibiting Th1 cytokine production of interleukin 2 (IL-2), gamma interferon (IFN-*γ*), and IL-2 receptor *α*-chain expression by engaging antigen-presenting cell- (APC-) bounds B7.1 (CD80) and B7.2 (CD86) ligands [10]. Functionally, CTLA-4 attenuates T-cell signaling by interference with intracellular signal transduction events, including TCR signaling, and reduced CTLA-4 expression and/or activity results in uncontrolled T-cell-associated autoimmunity and lymphoproliferative disease [7, 10]. In this regard, it was shown that CTLA-4 gene polymorphisms significantly influence the risk of autoimmune diseases, including Graves' disease, systemic lupus erythematosus, autoimmune hypothyroidism, celiac disease, Henoch-Schönlein purpura, type-1 diabetes [11–13], and BD [3], in many cohorts although this has not been studied in Egyptian BD.

It has been demonstrated that CTLA-4 is able to generate messenger RNA (mRNA) for two known isoforms: a full-length isoform (flCTLA-4) encoded by exon-1 (leader peptide), exon-2 (ligand binding domain), exon-3 (transmembrane domain), and exon-4 (cytoplasmic tail) and a soluble form (sCTLA-4), which lacks exon-3. sCTLA-4, originating from alternative splicing, results in the loss of a cysteine residue and is found in the serum as a soluble monomeric protein [14].

Several different polymorphisms in CTLA-4 have been described. Polymorphism at position 49 in exon-1 (+49 A/G, rs 231775) of CTLA-4 exerts differential functional effects on CTLA-4-driven downregulation of T-cell activation [15–17]. Considering the role of T cells in BD, the CTLA-4 49 A/G single nucleotide polymorphism could be associated with this disease, so in the present study, we aimed to investigate if +49 A/G single nucleotide polymorphism of CTLA-4 gene confers susceptibility to BD and its effect on the level of sCTLA-4 protein and their potential association to the main clinical features of the disease in Egyptian population.

## 2. Materials and Methods

### 2.1. Specimen Collection

We investigated the distribution of the CTLA-4 exon-1 position 49 A/G single nucleotide gene polymorphism (rs 231775) in 60 unrelated Egyptian patients with BD (22 women, 38 men; mean ± SD age, 34.8 ± 10.7 years), diagnosed according to the International Criteria for Behçet's Disease (ICBD) [18] in the period from January 2011 to June 2013, and 95 ethnically, age-, and sex-matched healthy individuals were taken as a control group. All participants enrolled in our study were collected from the in-patient and follow-up units of Rheumatology and Rehabilitation Departments and from the out-patient clinics of Ophthalmology Department, Zagazig University Hospitals. All participants provided written informed consent, and local ethics committee approval had been obtained.

### 2.2. Genomic DNA Extraction

Genomic DNA was extracted from a 5 mL sample of whole blood collected into EDTA. Extraction was performed using a commercial kit (QIAamp Blood Kit; Qiagen GmbH, Hilden, Germany) according to the manufacturer's instructions. DNA was stored at –20°C till the time of use.

### 2.3. Genotyping of the CTLA-4 Gene

For genotyping, PCR-restriction fragment length polymorphism was employed. The PCR was carried out in an AmpGene DNA thermal cycler 4800 and reaction mixtures of the total volume of 25 *μ*L included 10 *μ*g genomic DNA, 5 pmol of each primer (Promega, Madison, WI), and 1X PCR mix (Taq PCR Master Mix Kit, QIAGEN, GmbH, Hilden, Germany) containing 200 *μ*mol/L of each dNTP, 5 *μ*L of 10X reaction buffer, and 1.25 U Taq Gold Polymerase and 4 mmol/L MgCl_2_. Primers 5′-AAGGGCTCAGCTGAACCTGGT and 5′-CTGCTGAAACAAATGAAACCC (Fermentas, Berlin, Germany) were used to amplify the 152-bp DNA fragment of the CTLA-4 +49 A/G polymorphism. The PCR is followed by an overnight digestion with the restriction enzyme BstEII (Fermentas). All PCR products and the fragments were resolved by electrophoresis in a 3% agarose gel after staining with ethidium bromide.

### 2.4. Serum sCTLA-4 Levels

ELISA kits were used for measuring serum sCTLA-4 levels (Med System Diagnostic GmbH, Vienna, Austria) according to the manufacturer's protocol. A sCTLA-4 concentration of at least 1 ng/mL was considered positive.

### 2.5. Statistical Analysis

Data were analyzed with SPSS version 17.0 (statistical package for the Social Science, Chicago, IL). Data were expressed using descriptive statistic (mean and standard deviation and percentage). The frequencies for genotypes and alleles of the CTLA-4 gene for controls and BD patients were evaluated by chi-squared test. When the expected cell number was less than 5, Fisher's exact test was used. Hardy-Weinberg equilibrium analyses (HWP) were performed to compare observed and expected genotype frequencies using the X^2^ test. The associations between polymorphisms and BD as well as some clinical manifestation were estimated by the odds ratios (OR) and their 95% confidence intervals (CI). To compare the total serum sCTLA-4 levels between patients and controls, we used an independent* t*-test, while analyses for subgroups of genotypes, we used one-way analysis of variance (ANOVA) followed by least significance difference (LSD). A difference was considered statistically significant if *P* < 0.05.

## 3. Results

To determine whether CTLA-4 exon-1 gene polymorphism (+49 A/G, rs 231775) is associated with BD, in our subset of Egyptian patients, we genotyped normal controls as well as individuals with BD. The studied genotype and allele frequencies were in Hardy-Weinberg equilibrium in both BD and healthy control subjects. The demographic and clinical features of patients with BD are summarized in Table 1.

The distribution of genotypes and allele frequencies in BD and controls is presented in Table 2. Among patients with BD, there were significantly higher frequency of A/A genotype in the patient group when compared with controls (51.7% versus 22.1%; *P* < 0.001; OR = 6.643 and CI (95%) = 2.58–17.10). Therefore, the gene frequency of the A allele was higher in BD patients than in controls (69.2% versus 42.1%, resp.; *P* < 0.001; OR = 3.084 and CI (95%) = 1.90–4.99) in our population.

In the present study, there were no significant differences observed in the genotype or allele frequencies distribution between male and female patients with BD; see Table 3.

When the allele and genotype frequencies were analyzed according to the clinical features of BD (see Table 4), we found that both the A/A genotype and A allele frequencies were significantly higher in patients with positive skin pathergy test in comparison to patients without positive skin pathergy test (*P* value = 0.018; odds ratio = 8.63; CI (95%) = 1.44–51.7) (*P* value = 0.009; odds ratio = 2.89; CI (95%) = 1.3–6.4), retinopathy (*P* value = 0.041; odds ratio = 6.31; CI (95%) = 1.075–36.9) (*P* value = 0.027; odds ratio = 2.44; CI (95%) = 1.1–5.4), and vasculitic manifestations (*P* value = 0.028; odds ratio = 7.33; CI (95%) = 1.2–43.4) (*P* value = 0.014, odds ratio = 2.72; CI (95%) = 1.2–6.03).

In this study, BD patients were found to have significantly lower levels of serum CTLA-4 concentration than controls (1.76 ± 0.19 versus 1.91 ± 0.30; *P* < 0.0007); see Table 5. Furthermore, when the sCTLA-4 concentrations in sera of patients with different genotypes were compared together, there was a significant lower level of sCTLA-4 concentrations in patients with A/A genotype in comparison to other genotypes groups.

## 4. Discussion

The present study analyses the association between the CTLA-4 exon-1 49 A/G polymorphism with BD susceptibility and clinical manifestations. Allele and genotype frequencies of CTLA-4 49 A and A/A were significantly increased in BD patients in comparison to healthy controls, suggesting that this genotypic variant seems to confer the susceptibility to BD, reducing the expression of sCTLA-4 protein in BD patients, and significantly associated with vasculitic manifestations of the disease.

Our results demonstrated that BD patients have significantly higher frequencies of A/A genotype and A allele in comparison to the control group. This is in accordance with ben Dhifallah et al. [19] study that was conducted on Tunisian population. In addition, in another study by Gunesacar et al. [20] on Turkish population, G/G genotype was significantly decreased more in BD patients than the controls, negatively associated with BD, and plays a protective role from BD.

Meanwhile, our results disagree with the meta-analysis study of Lee and Song et al. [21] and Zhang et al. [22] as well as with multiple previous studies conducted on different ethnic populations like Chinese [23], Korean [24], and UK and Middle East populations [25]. This inconsistency may be due to the racial/ethnic differences in allele frequencies, population differences, environmental factors (microbial loading), and limited number of study participants or due to the wide differences of the clinical manifestations in BD patients.

We are also in discordance more with the results of Sallakci et al. [3], which was conducted on another smaller Turkish population, than those of Gunesacar et al. [20], where there were absent associations between the CTLA-4 +49 A/G polymorphism and susceptibility to BD, although they observed that BD patients with ocular involvement and erythema nodosum-like lesions had higher frequencies of both the A allele and the AA genotype at position 49 of exon-1, so they consider CTLA-4 as a disease-modifying rather than a susceptibility gene for BD.

Although our results are in accordance with both previous results [3, 20] in regard to absence of significant statistical differences in the genotype or allele frequencies distribution between males and females of BD patients, they are in discordance with those of ben Dhifallah et al. [19], who found the A allele more prevalent in BD males than females.

In the present study, although there was no significant association between genotypes and disease manifestations as arthritis, uveitis, GIT, and CNS manifestations (data not shown in Table 5), A/A genotype and A allele frequencies were significantly higher in patients with retinopathy and positive skin pathergy test. This is in accordance with results of Sallakci et al. [3] but in opposition to the results found in other previous studies [19, 20, 24, 25], where there was no significant association between the studied polymorphism and clinical manifestations of BD. We thought that discrepancy between our results and those of Gunesacar et al. [20] may be due to negative results of A allele and A/A genotype which they reported while being elevated in our group of patients. On the other hand, it was mentioned that CTLA-4 polymorphisms have been associated with strong autoantibody component diseases like SLE and not with T-cell-mediated diseases like RA and BD [25].

Also, we found that vasculitic manifestations in BD patients showed significant association with A/A and A allele. The presence of anti-CTLA-4 antibody has been reported in a fraction of Behçet's disease patients that might be possibly involved in abnormal T-cell responses. This antibody might be produced only as a secondary phenomenon of recurrent T-cell activation in Behçet's disease [26]. These increased anti-CTLA-4 antibodies may suppress Th1 cell function and may cause a relative Th2 cell activation, leading to immune complex-mediated vasculitis in patients with BD [3].

We can suggest that the coincidental flaring of some clinical manifestations of the disease in patients with A/A genotype raises the possibility that this genotype is a susceptible and disease-modifying gene for BD.

Many studies have shown higher levels of Th1-type proinflammatory cytokines, including TNF-*α*, interferon- (IFN-) c, IL-2, IL-12, and IL-18, and others demonstrated high levels of IL-1, IL-6, IL-8, IL-10, soluble IL-2 receptor (sIL-2R), IFN-c, and TNF-*α* in BD, indicating mixed Th1- and Th2-type immune response responsible for the higher degree of immune activity in BD [3, 25]. Moreover, it was proven that signaling through CTLA-4 downregulates T helper- (Th-) 1 cell proliferation and also delivers a positive signal to Th-2 cell activation [3].

In the present study, there were significantly decreased sCTLA-4 concentrations in BD patients in comparison to the controls being in concordance with Park et al. [24] who found that the mean serum sCTLA-4 concentration in BD patients was significantly lower than that in controls, concluding that decreased level of sCTLA-4 might result in enhanced activation and proliferation of T cells, which could lead to the development of chronic inflammatory diseases like BD [24]. However, this result is partially contradictory to that of Sim et al. [27] who reported a reduced expression of CTLA-4 in CD4+ T cells in patients with active Behçet's disease, with no difference in the production of sCTLA-4, leading to subsequent increase in Th1 cell proliferation and a tendency to the development of Behçet's disease. In addition, when comparing between the different genotype groups of BD patients, there was significant decreased sCTLA-4 level in sera of patients with A/A genotype in comparison to other genotypes groups and to control group (*P* value < 0.0001). On the contrary, Park et al. [24] found that the serum sCTLA-4 levels in BD patients with the CTLA-4 +49 G allele were significantly lower than those in healthy controls. We can explain that by the abnormal translation of sCTLA-4 and/or intracellular trafficking release of sCTLA-4 or posttranslation protein modification, like phosphorylation, glycosylation, or ubiquitination which plays a key role in protein degradation [28], may occur in these patients but this needs further investigations.

Our study faced a number of limitations, mainly the small number of patients specimen that makes the classification into subgroups according to clinical manifestations difficult to interpret more accurately, but this could be due to the rarity of such an autoimmune disease [29] especially in Egypt (7.6/10^5^ population) when compared with other countries [30], as well as the delayed contact of BD patients with rheumatologists, which makes exact incidence and prevalence of BD in Egypt not well known.

## 5. Conclusion

CTLA-4 49 A/A genotype could confer susceptibility to BD. It is correlated significantly with some clinical manifestations of the disease through reducing the expression of serum sCTLA-4 in an Egyptian population, but these findings need further confirmatory studies on larger cohorts.

## Figures and Tables

**Table 1:** Demographic characteristics of patients with Behçet's disease and frequency of clinical manifestations.

Variables	Values
Sex (male : female)	38 : 22
Age (mean ± SD), years	34.8 ± 10.7
Disease duration, years	7.2 ± 3.9

Clinical characteristics	Number of cases, *n* (%)

Major symptoms	
Oral ulcer	60 (100.0)
Genital ulcer	41 (68.3)
Skin lesions	12 (20.0)
Uveitis	19 (31.7)
Retinopathy	35 (58.3)
Minor symptoms	
Arthritis	25 (41.7)
Gastrointestinal involvement	11 (18.3)
Central nervous system involvement	26 (40.3)
Vasculitis	36 (60.0)
Headache	21 (35.0)
Skin pathergy test	38 (63.3)

**Table 2:** Comparisons of allele and genotype frequencies of CTLA-4 +49A/G polymorphism in patients with Behçet's disease and healthy controls.

CTLA-4 49 A	Genotypes frequency, *n* (%)			Alleles frequency, *n* (%)	
	AA	AG	GG	A	G
Patients (*n* = 60)	31 (51.7)	21 (35.0)	8 (13.3)	83 (69.2)	37 (30.8)
Controls (*n* = 95)	21 (22.1)	38 (40.0)	36 (37.9)	80 (42.1)	110 (57.9)
*P* value	<0.001*	0.056		<0.001*	
OR	6.643	2.487		3.084	
CI (95%)	2.58–17.10	0.98–6.32		1.90–4.99	

*P value is significant <0.05, *χ* ^2^: chi-square, OR: odds ratio, and CI: confidence interval.

**Table 3:** CTLA-4 49A/G allele and genotype frequencies distribution between male and female patients with Behçet's disease (BD).

CTLA-4 49 A	Genotypes frequency, *n* (%)			*χ* ^2^ (*P* value)	Alleles frequency, *n* (%)		*χ* ^2^ (*P* value)
	AA	AG	GG		A	G	
Males (*n* = 38)	20 (52.6)	13 (34.2)	5 (13.2)	0.858 (0.651)	53 (69.3)	23 (30.7)	0.469 (0.312)
Females (*n* = 22)	9 (40.9)	10 (45.5)	3 (13.6)		28 (63.6)	16 (36.4)	

**Table 4:** CTLA-4 genotypes and allele frequencies in relation to some clinical manifestations of Behçet's disease in Egyptian patients.

CTLA-4 49 A	Genotypes frequency, *n* (%)			Alleles frequency, *n* (%)	
	AA	AG	GG	A	G
Skin pathergy					
With	23 (60.5)*	13 (34.2)	2 (5.3)	59 (77.6)^#^	17 (22.4)
Without	8 (36.4)	8 (36.4)	6 (27.3)	24 (54.5)	20 (45.5)
Retinopathy					
With	21 (60.0)^†^	12 (34.3)	2 (5.7)	54 (77.1)^¶^	16 (22.9)
Without	14 (40.0)	9 (36.0)	6 (24.0)	29 (58.0)	21 (42.0)
Vasculitis					
With	22 (61.1)^*♯*^	12 (33.3)	2 (5.6)	56 (77.8)^§^	16 (22.2)
Without	9 (37.5)	9 (37.5)	6 (25.0)	27 (56.2)	21 (43.8)

*P value = 0.018; odds ratio = 8.63 in patients with versus without skin pathergy test.

^#^ *P* value = 0.009; odds ratio = 2.89 in patients with versus without skin pathergy test.

^†^ *P* value = 0.041; odds ratio = 6.31 in patients with versus without retinopathy.

^¶^ *P* value = 0.027; odds ratio = 2.44 in patients with versus without retinopathy.

^*♯*^ *P* value = 0.028; odds ratio = 7.33 in patients with versus without vasculitic manifestations.

^§^ *P* value = 0.014; odds ratio = 2.72 in patients with versus without vasculitic manifestations.

**Table 5:** Serum CTLA-4 levels (ng/mL) mean ± SD according to CTLA-4 genotypes frequencies in both patients and control groups.

	CTLA-4 49 A genotypes frequency			*P* value
	AA	AG	GG	
Controls (*n* = 95)	1.84 ± 0.16	1.90 ± 0.14	1.95 ± 0.28	0.135
Patients (*n* = 60)	1.49 ± 0.23	1.89 ± 0.15	1.92 ± 0.30	0.001
*P* value	<0.0001	0.674	0.528	
